# Efficacy and safety of cisplatin for the management of adult patients with oral cancer: A protocol for systematic review

**DOI:** 10.1097/MD.0000000000018210

**Published:** 2019-12-20

**Authors:** Yao Feng, Dian-song Yang, Hai-bo Tang, Yuan-sheng Ding, Xiao-guang Li

**Affiliations:** a First Unit of Dental Pulp Disease Department; b Department of Radiation; c Department of Orthodontics, Second Affiliated Hospital of Jiamusi University, Jiamusi, China.

**Keywords:** cisplatin, efficacy, oral cancer, safety

## Abstract

**Background:**

Cisplatin is often used for the treatment of oral cancer (OC). However, there are inconsistent results. Thus, this study plans to systematically assess the clinical efficacy and safety of cisplatin for adult patients with OC.

**Methods:**

We will search for PUBMED, EMBASE, Cochrane Library, AMED, Cumulative Index to Nursing and Allied Health Literature, Chinese Biomedical Literature Database, and China National Knowledge Infrastructure. All of them will be searched from the construction of each database up to the present with no restrictions of language and publication status. The data analysis will be conducted using RevMan 5.3 software to assess the efficacy and safety of cisplatin for adult patients with OC.

**Results:**

This study will summarize the most recent high-quality evidence and will provide helpful information about the efficacy and safety of cisplatin for adult patients with OC.

**Conclusion:**

The findings of this study will provide convinced evidence of cisplatin for adult patients with OC, and provide recommendations for clinical practice.

**Systematic review registration:**

PROSPERO CRD42019156558.

## 1 Introduction

Oral cancer (OC) is one of the most common cancers worldwide, and has a 5-year survival rate of around 50%.^[1–4]^ It has been reported about 354,864 new cases of OC were identified, and 177,384 people died from OC globally in 2018.^[5]^ Although new patients have been diagnosed increasingly, most patients are still diagnosed at late and advanced stages at the initial diagnosis.^[6–9]^ Due to its anatomic location, patients with such disorder often experience very poor quality of life, impairment of most vital functions, as well as their appearances.^[10–12]^ Thus, it is very urgent to treat such condition timely and effectively.

Cisplatin has been reported to treat a variety of cancers effectively, especially for OC.^[13–16]^ However, there is still no systematic evidence of cisplatin for the treatment of adult patients with OC.^[17–21]^ Therefore, this study will assess the efficacy and safety of cisplatin for the treatment of adult patients with OC systematically.

## 2 Methods

### 2.1 Inclusion criteria

#### 2.1.1 Types of studies

Any randomized controlled trials (RCTs) using cisplatin to treat adult patients with OC will be included, regardless of blinding, and allocation concealment.

#### 2.1.2 Types of patients

This study will include adult patients (aged more than 18 years old) who were diagnosed as OC without limitations of gender, race, and duration of disease.

#### 2.1.3 Types of interventions

The patients in the experimental group received cisplatin treatment, regardless its forms and dosage.

The patients in the control group received any routine treatments, such as radiation therapy, surgery, and any others, except cisplatin.

#### 2.1.4 Type of outcome measurements

The primary outcomes are overall survival and pathological complete response. The secondary outcomes are recurrence-free survival; disease-free survival; quality of life, as measured by 36-Item Short Form Health Survey or any related tools; and toxicities.

### 2.2 Data sources and search strategy

#### 2.2.1 Electronic searches

We will carry out comprehensive search from the construction of each database up to the present with no restrictions of language and publication status from following electronic databases: PUBMED, EMBASE, Cochrane Library, AMED, Cumulative Index to Nursing and Allied Health Literature, Chinese Biomedical Literature Database, and China National Knowledge Infrastructure. The search strategy for PUBMED is showed in Table 1. We also plan to build similar search strategies for other electronic databases.

**Table 1:**
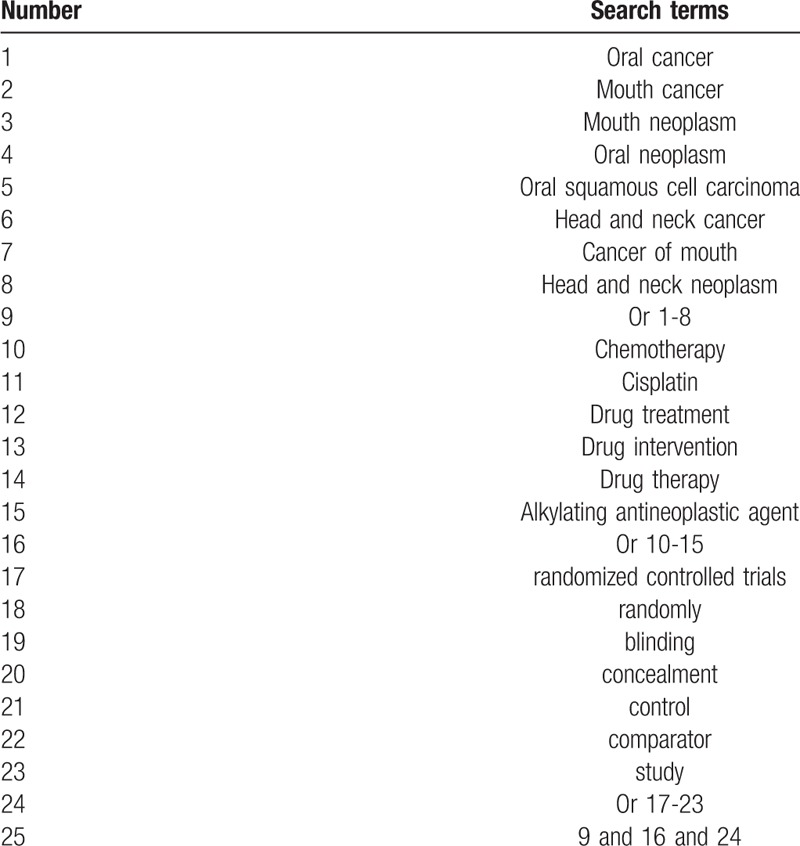
Search strategy for PUBMED.

#### 2.2.2 Other resources

We will also check dissertations, ongoing trials, and reference lists of identified studies to avoid losing any potential studies.

### 2.3 Data collection and analysis

#### 2.3.1 Study selection

All identified papers will be checked through scanning their titles and abstracts by 2 independent authors. Then, we will exclude all irrelevant studies. The rest literatures will be read by full texts to further judge if they meet all inclusion criteria. Any excluded studies will be recorded with clear reasons. Any divergences between 2 authors will be settled down by a third author through discussion. The study selection will be presented in the flowchart.

#### 2.3.2 Data collection process

Two authors will independently carry out data collection using pre-designed data extraction form. It consists of study characteristics (such as first author name, publication date, title, country, et al), patient characteristics (such as race, age, gender, inclusion criteria, et al), study methods (such as sample size calculation, methods of randomization, blind, et al), study treatments (such as intervention types, frequency, dosage, et al), all outcomes, safety, and other relevant information. When there is any missing or insufficient information, we will contact original authors by email to require them. Any divergences between 2 authors will be resolved by a third author via discussion.

### 2.4 Risk of bias assessment

Two authors will independently evaluate risk of bias of each included trial using Cochrane risk of bias tool according to the specific Guidelines of Cochrane Handbook for Systematic Reviews of Interventions. It has 7 detailed items, and each one is further assessed as “low risk of bias”, “unclear risk of bias”, and “high risk of bias”. Any disagreements will be solved by another author via discussion.

### 2.5 Assessment of heterogeneity

We will estimate heterogeneity among eligible studies using *I*^*2*^ statistic test. The value of *I*^*2*^ ≤ 50% refers low heterogeneity, while the value of *I*^*2*^ > 50% exerts significant heterogeneity.

### 2.6 Subgroup analysis

If there are sufficient studies for data pooling with substantial heterogeneity, we will perform subgroup analysis to explore reasons for such obvious heterogeneity according to the different risk of bias, treatments, comparators, and outcome measurements.

### 2.7 Sensitivity analysis

We will consider conducting sensitivity analysis to check stability of pooled results by eliminating low quality studies.

### 2.8 Reporting bias

If at least 10 included RCTs are entered in this study, we will perform funnel plots to check any potential reporting bias.^[22]^

### 2.9 Statistical analysis

RevMan 5.3 software will be used to carry out data analysis. For continuous data, mean difference or standardized mean difference and 95% confidence intervals (CIs) will be expressed. For dichotomous data, risk ratio and 95% CIs will be calculated. Heterogeneity across included trials will be identified using *I*^*2*^ statistic. *I*^2^ ≤ 50 indicates included studies have homogeneity, and a fixed-effects model will be selected. *I*^2^ > 50% means eligible studies have significant heterogeneity, and a random-effects model will be adopted. If there is homogeneity among more than 2 studies with similar treatments, controls, and outcomes, we will perform meta-analysis. If there is substantial heterogeneity, subgroup analysis will be operated. If there is still obvious heterogeneity after subgroup analysis, outcome data are not available for quantitative analysis, and we will report study results by qualitative description.

### 2.10 Ethics and dissemination

This study will not need research ethic, because we will use individual patient data. It is expected to be published on a peer-reviewed journal.

## 3 Discussion

OC is a devastating disease, which can result in highly morbidity and mortality in both males and females. Although promising advancements in its management approaches available, the overall efficacy is still not satisfied. Fortunately, previous studies have reported that cisplatin can benefit adult patients with OC. However, there is still not systematic review to address it. Therefore, this study will assess the efficacy and safety of cisplatin for the treatment of adult patients with OC. The results of this study will provide helpful evidence for OC treatment, and helpful recommendation for clinical practice.

## Author contributions

**Conceptualization:** Yao Feng, Dian-song Yang, Hai-bo Tang, Yuan-sheng Ding, Xiao-guang Li.

**Data curation:** Dian-song Yang, Hai-bo Tang, Yuan-sheng Ding, Xiao-guang Li.

**Formal analysis:** Yao Feng, Hai-bo Tang, Yuan-sheng Ding.

**Funding acquisition:** Yao Feng.

**Investigation:** Yao Feng, Dian-song Yang, Xiao-guang Li.

**Methodology:** Dian-song Yang, Hai-bo Tang, Yuan-sheng Ding, Xiao-guang Li.

**Project administration:** Yao Feng, Dian-song Yang.

**Resources:** Hai-bo Tang, Yuan-sheng Ding, Xiao-guang Li.

**Software:** Dian-song Yang, Hai-bo Tang, Yuan-sheng Ding, Xiao-guang Li.

**Supervision:** Yao Feng.

**Validation:** Yao Feng, Dian-song Yang, Hai-bo Tang, Yuan-sheng Ding, Xiao-guang Li.

**Visualization:** Yao Feng, Hai-bo Tang, Yuan-sheng Ding, Xiao-guang Li.

**Writing – original draft:** Yao Feng, Yuan-sheng Ding, Xiao-guang Li.

**Writing – review & editing:** Yao Feng, Dian-song Yang, Hai-bo Tang, Yuan-sheng Ding, Xiao-guang Li.
